# Nuclear Lipid Microdomain as Resting Place of Dexamethasone to Impair Cell Proliferation

**DOI:** 10.3390/ijms151119832

**Published:** 2014-10-31

**Authors:** Samuela Cataldi, Michela Codini, Giacomo Cascianelli, Sabina Tringali, Anna Rita Tringali, Andrea Lazzarini, Alessandro Floridi, Elisa Bartoccini, Mercedes Garcia-Gil, Remo Lazzarini, Francesco Saverio Ambesi-Impiombato, Francesco Curcio, Tommaso Beccari, Elisabetta Albi

**Affiliations:** 1 Laboratory of Nuclear Lipid BioPathology, Crabion, 06074 Perugia, Italy; E-Mails: samuelacataldi@libero.it (S.C.); giacomocascianelli@tiscali.it (G.C.); andrylazza@gmail.it (A.L.); info@crabion.it (A.F.); infoe@crabion.it (E.B.); remo30@libero.it (R.L.); 2 Department of Pharmaceutical Science, University of Perugia, 06100 Perugia, Italy; E-Mails: codini@virgilio.it (M.C.); tommaso.beccari@unipg.it (T.B.); 3 Laboratory of Clinical Pathology, 96011 Augusta-Siracusa, Italy; E-Mails: info@lab-tringali-s.com (S.T.); annatringali69@gmail.com (A.R.T.); 4 Department of Biology, University of Pisa, 56127 Pisa, Italy; E-Mail: mercedes.garcia@unipi.it; 5 Department of Clinical and Biological Sciences, University of Udine, 33100 Udine, Italy; E-Mails: ambesis@me.com (F.S.A.-I.); curcio@uniud.it (F.C.)

**Keywords:** dexametasone, lymphoma, nuclear lipid microdomains, sphingomyelin, sphingomyelinase, sphingomyelin-synthase

## Abstract

The action of dexamethasone is initiated by, and strictly dependent upon, the interaction of the drug with its receptor followed by its translocation into the nucleus where modulates gene expression. Where the drug localizes at the intranuclear level is not yet known. We aimed to study the localization of the drug in nuclear lipid microdomains rich in sphingomyelin content that anchor active chromatin and act as platform for transcription modulation. The study was performed in non-Hodgkin’s T cell human lymphoblastic lymphoma (SUP-T1 cell line). We found that when dexamethasone enters into the nucleus it localizes in nuclear lipid microdomains where influences sphingomyelin metabolism. This is followed after 24 h by a cell cycle block accompanied by the up-regulation of cyclin-dependent kinase inhibitor 1A (*CDKN1A*), cyclin-dependent kinase inhibitor 1B (*CDKN1B*), growth arrest and DNA-damage 45A (*GADD45A*), and glyceraldehyde 3-phosphate dehydrogenase (*GAPDH*) genes and by the reduction of signal transducer and activator of transcription 3 (STAT3) and phospho signal transducer and activator of transcription 3 (phoshoSTAT3) proteins. After 48 h some cells show morphological changes characteristic of apoptosis while the number of the cells that undergo cell division and express B-cell lymphoma-2 (Bcl-2) is very low. We suggest that the integrity of nuclear lipid microdomains is important for the response to glucocorticoids of cancer cells.

## 1. Introduction

Glucocorticoids (GCs) are used in the treatment of many haematological malignancies including leukaemia, lymphoma and multiple myeloma [1,2,3]. It is known that action of GCs is initiated by, and strictly dependent upon, the interaction of the drug with its receptor followed by its translocation into the nucleus where modulates gene expression. The removal within the first 24 h of GC treatment prevents cell death in acute leukemia lymphoblastic cells [4]. The glucocorticoid receptor (GR) has been cloned, sequenced and found to be organized into a discrete series of domains which mediate the receptor functions [1]. GR belongs to the nuclear receptor superfamily of transcription factors that initiate specific genetic programs important in all physiological processes, including development, metabolism, differentiation, and growth [5]. After ligand activation, GR translocates to the nucleus and dimerizes on specific response elements within the genome [6]. Upon target binding, nuclear receptors are then able modulate gene output by the recruitment of cofactors and transcriptional machinery [7]. In particular, upon DNA binding by the receptor, different cofactors are believed to manipulate the chromatin structure, providing open and accessible DNA for the transcriptional machinery [8]. Trafficking of the GR between cellular compartments is controlled by multiple proteins, including FK506-binding proteins 51 (FKBP51, FKBP5) and 52 (FKBP52, FKBP4), heat shock protein 90 (Hsp90), cyclophilin 40, dynein and dynamitin [9]. Therefore, how Dexamethasone (Dex) arrives from the cell membrane to the nucleus and how this traffic is regulated have been widely studied [1,2,3,4,5,6,7,8,9], but until now it is not known where the drug localizes at the nuclear level.

Control of the cellular cycle is regulated by cyclins and cyclin-dependent kinases (CDKs). The activity of these enzymes is restricted by the inhibiting action of CDK inhibitors (CDKN). Cyclin-dependent kinase inhibitor 1A (CDKN1A, p21) is induced in response to DNA damage through the activity of the protein 53 (p53) tumor suppressor protein and mediates cell cycle arrest in growth 1 (G1) and growth 2 (G2) phases by acting at the G1/synthesis (S) and G2/mitosis (M) cell cycle checkpoints [10]. Cyclin-dependent kinase inhibitor 1B (CDKN1B, p27) plays a critical role in regulating G1/S transition of the cell cycle [11]. Growth arrest and DNA-damage 45A (*GADD45A*) is one of the DNA-damage checkpoint genes that upon various kinds of stress maintains genomic integrity in many cell types, through promoting cell death, cell cycle arrest, and DNA repair [12]. Signal transducer and activator of transcription 3 (STAT3) activity stimulates cell-cycle progression, cell proliferation, and survival [13] after its cytoplasm-nucleus translocation [14]. B-cell lymphoma-2 (Bcl-2) is an inner mitochondrial membrane protein that extends the survival of the cells by blocking programmed cell death [15].

Sphingomyelin (SM) is a lipid highly represented inside the nucleus and its amount changes in cell proliferation, differentiation and/or apoptosis thanks to the presence of the enzymes for its metabolism [16]. In fact, in the nucleus, SM can be catabolized by sphingomyelinase (SMase) to produce ceramide and phosphorylcholine (PPC) [17], can be used as source of PPC for phosphatidylcholine (PC) synthesis by reverse-SM-synthase [18] and can be synthesized by SM-synthase by using PC as source of PPC [19]. Nuclear SM is associated with perichromatin fibrils that are *in situ* expressions of nascent pre-mRNA transcripts [20]. In inner nuclear membrane SM links cholesterol (CHO) to form nuclear lipid microdomains (NLMs) that represent an attachment site for active chromatin during cell proliferation [21], act as platform for the transcription process [22,23], and act as platform for Vitamin D3–Vitamin D3 receptor interaction, inducing embryonic hippocampal cell differentiation [24].

We aimed to study the localization of the Dex in NLM after its translocation inside the nucleus and its effect in human lymphoblastic lymphoma T cell growth.

## 2. Results

### 2.1. Non-Hodgkin’s T Cell Human Lymphoblastic Lymphoma Cell Growth Is Suppressed by Dexamethasone

**Figure 1:**
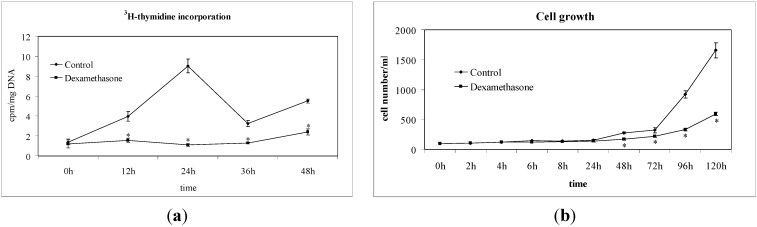
Effect of Dexamethasone on DNA synthesis and cell growth. (**a**) Incorporation of ^3^H-thymidine in non-Hodgkin’s T cell human lymphoblastic lymphoma cell line (SUP-T1) cells. Cells were cultured for 48 h without (C = control sample) or with 100 Dex. 1 µCi ^3^H-tymidine was added to the medium 2 h before the analysis. The radioactivity was evaluated on extracted nucleic acid in each preparation. The data are expressed as cpm/mg DNA and represent the mean ± S.D. of 3 independent experiments performed in duplicate. (Significance, * *p* < 0.001 *versus* 0 h); (**b**) Count of cell number. The data are expressed as cell number/mL and represent the mean ± S.D. of 3 independent experiments performed in duplicate. (Significance, * *p* < 0.001 *versus* control sample).

**Figure 2:**
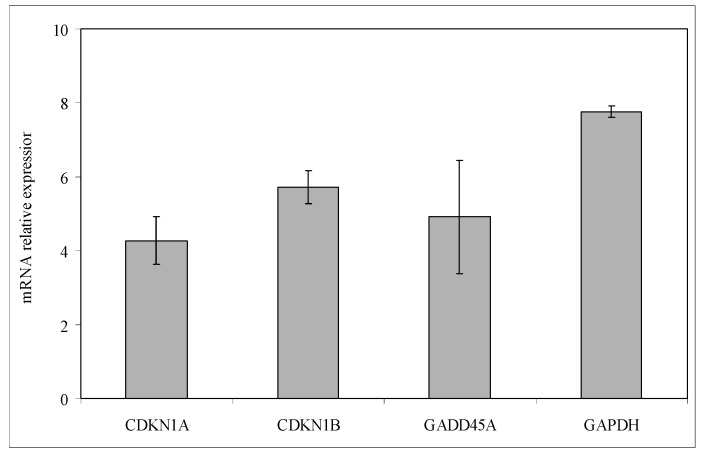
Effect of Dexamethasone on cyclin-dependent kinase inhibitor 1A (*CDKN1A)*, cyclin-dependent kinase inhibitor 1B (*CDKN1B)*, growth arrest and DNA-damage 45A (*GADD45A)* and glyceraldehyde 3-phosphate dehydrogenase (*GAPDH*) and expression. RTqPCR analysis was performed in control and experimental SUP-T1 cells collected after 24 h from Dex treatment. GAPDH has long been used as a default reference gene in quantitative mRNA profiling experiments; however, since its expression varied in cancer as for many other genes, was preferred absolute quantification and untreated SUP-T1 cells were used for *C*_T_ comparison [28]. In ordinate, mRNA relative expression = mRNA of treated cells/mRNA of controls. Data are expressed as the mean ± S.D. of 3 independent experiments performed in three PCR replicates.

**Figure 3:**
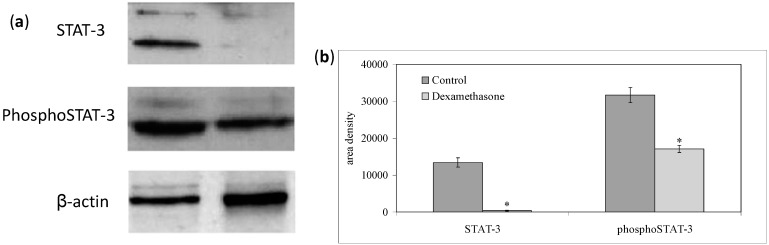
Effect of Dexamethasone on signal transducer and activator of transcription 3 (STAT3) and phospho signal transducer and activator of transcription 3 (phosphoSTAT3). (**a**) Immunoblots of proteins were probed with anti-STAT3 and anti-phospho-STAT3 and visualized by enhanced chemiluminescence (ECL). Apparent molecular weight for STAT3, phosphoSTAT3 and β-actin were 90, 92 and 43 KDa, respectively; (**b**) The area density was evaluated by densitometry scanning and analysis with Scion Image, the data represent the mean ± S.D. of three experiments performed in duplicate. (Significance, * *p* < 0.001 *versus* control sample).

**Figure 4:**
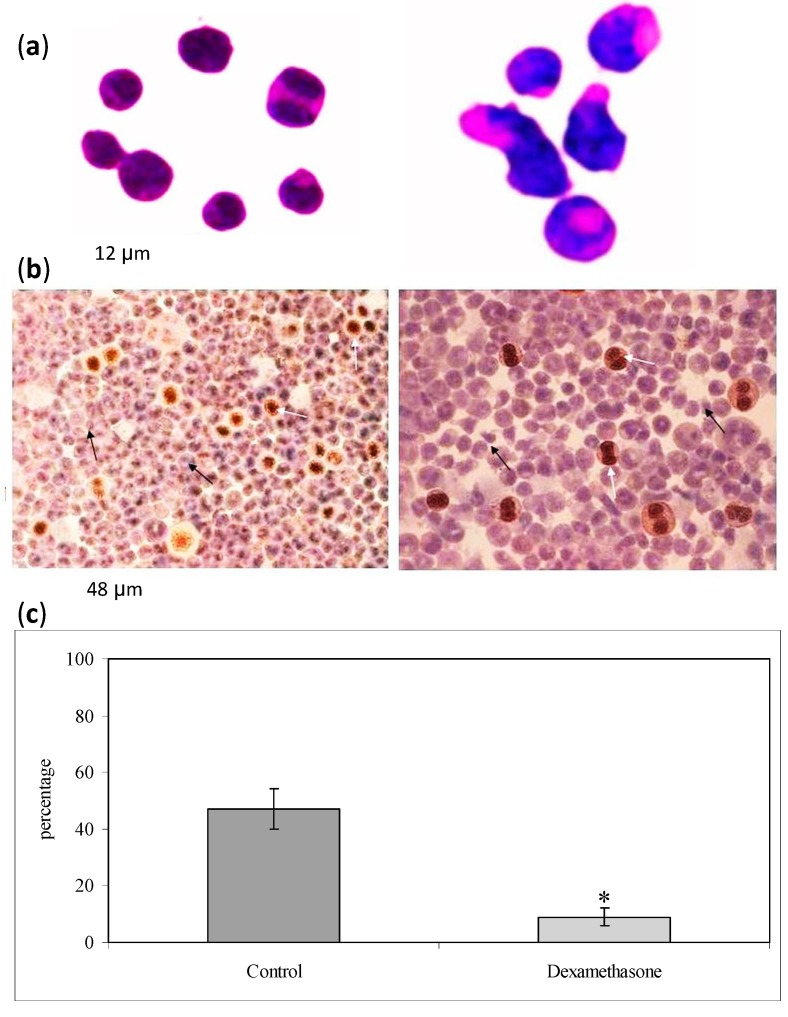
Effect of Dexamethasone on Cell Morphology and B-cell lymphoma-2 (Bcl-2) expression. The image analysis of morphology and Bcl-2 positivity was performed by using inverted microscopy EUROMEX FE 2935 (ED Amhem, The Netherlands) equipped with a CMEX 5000 camera system. (**a**) Rappresentative example of cells cultured in the absence (C = control) or in the presence of Dex for 48 h. Hematoxylin-eosin staining, 40× magnification. The images were highly contrasted to cancel the background and highlight the characteristics of the cells; (**b**) Immunohystochemical analysis Bcl-2. The cells were analyzed after 48 h of culture in the absence (C) or presence of Dex. White arrows indicate examples of normal (C) or apoptotic (Dex) cells, black arrows indicate Bcl-2 positive cells (10× magnification); (**c**) The intensity of immunostaining was evaluated. The findings were classified as follows: (−), no reactive cells; (+), low positive cells; (2+), medium positive cells; (3+), strong positive cells. Only the 3+ cells, with intense immunostaining, were considered for quantification. The data are expressed as percentage of total cells that resulted Bcl-2 3+ positive and represent the mean ± S.D. of 3 independent experiments performed in duplicate. (Significance, * *p* < 0.001 *versus* control sample).

We first investigated the effect of Dex on the non-Hodgkin’s T cell human lymphoblastic lymphoma cell line (SUP-T1). The results showed that in the control cells the specific activity of the DNA, calculated as cpm/µg DNA, increased at 12 h, and reached a peak at 24 h, which corresponded to the S phase of the cell cycle (Figure 1a). Dex treatment caused a strong decrease of ^3^H-thymidine already detectable at 12 h; the value remained constant until 48 h, when it slightly increased (Figure 1a). At this time, the number of control cells was 278 ± 13 number/mL and that of Dex-treated cells was 168 ± 16. The difference in cell growth between control and experimental samples increased in time (Figure 1b). To highlight the inhibition of the cell cycle, we study the gene expression of *CDKN1A*, *CDKN1B*, *GADD45A* [10,11,12]. Glyceraldehyde 3-phosphate dehydrogenase (GAPDH) has long been used as a default reference gene in quantitative mRNA profiling experiments. It is a ubiquitous enzyme that catalyzes the sixth step of glycolysis and plays a role in the control of gene expression and redox post-translational modifications [25]. Thus, its expression varied in response to a range of pathophysiological variables [25,26]. Our results showed that Dex treatment increased 8 times GAPDH expression (Figure 2). On the other hand, treatment with drugs changes the expression of many genes and a specific study is needed to identify the housekeeping genes [27]. Since at this moment no specific studies have been done on SUP-T1 cells treated with Dex to identify housekeeping gene, we evaluated mRNA expression of Dex-treated cells in relation to mRNA of control cells, according to Schmittgen and Livak [28]. Our results showed that the massive block of proliferation was accompanied, at 24 h from Dex incubation, by an up-regulation of CDKN1A, CDKN1B and GADD45A equal to 4.27, 5.71 and 4.91 times respectively, in comparison with control samples (Figure 2). Since it has been demonstrated that Dex inhibited phospho signal transducer and activator of transcription 3 (phosphoSTAT3) [29], we performed experiments of immunoblotting after 24 h of drug incubation showing a strong decrease of STAT3 and phosphoSTAT3 content (Figure 3a,b). As control for immunoblotting technique we used β-actin, normally used, but it increased strongly (Figure 3a). On the other hand it is known that Dex acted on actin networks [30]. Therefore it was not a good control but its increase was an indication that the reduction of STAT3 and phosphoSTAT3 was not due to the experimental defect. We next wondered whether later, at 48 h after Dex treatment, there might be changes in cell morphology and anti-apoptotic Bcl-2 protein content, up-regulated in T-cell acute lymphoblastic leukemia [31]. Hematoxylin-eosin staining showed round cells with nuclei intensely colored and cells ready for division. In the experimental sample it was possible to notice a change in the shape of the cells (Figure 4a). As shown in Figure 4b, Dex-treated cells were reduced in number but were bigger than controls with a small amount of the cells with altered morphology, similar to that indicated in Figure 4a. The percentage of Bcl-2 positive cells was reduced 5 times; the only positive cells were those that were ready for starting mitosis (Figure 4b,c).

### 2.2. Dexamethasone Localizes in Nuclear Lipid Microdomains

**Figure 5:**
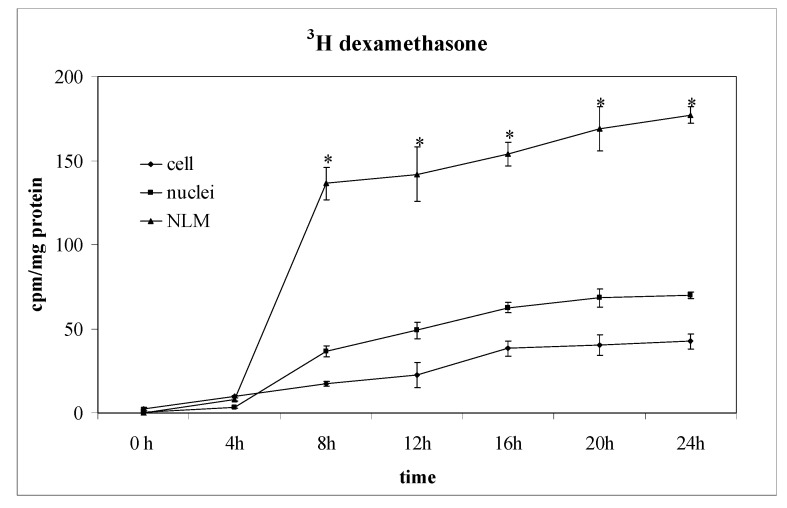
^3^H Dexamethasone content in cell homogenate, nuclei and nuclear lipid microdomains of SUP-T1 cells. Cells were incubated in the presence of ^3^H-Dex and cell homogenate, nuclei and nuclear lipid microdomains were prepared at different times and the radioactivity was measured as reported under “Materials and Methods” section. The data are expressed as cpm/mg protein and represent the mean ± S.D. of 3 experiments performed in duplicate. (Significance, * *p* < 0.001 *versus* cell or nuclei samples).

**Figure 6:**
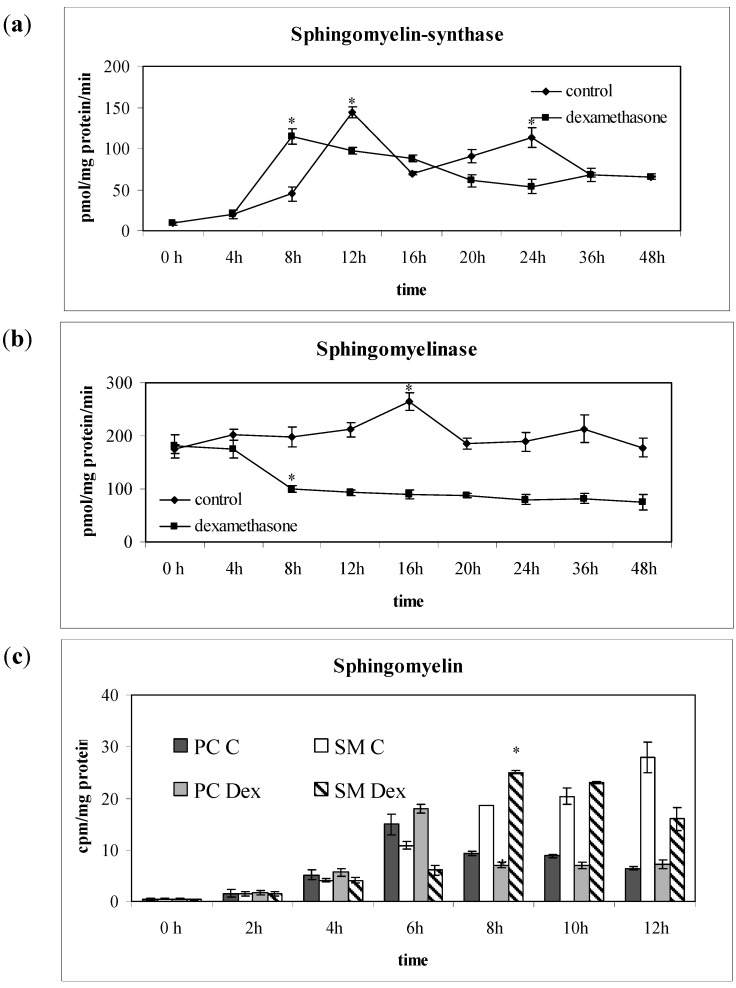
Effect of Dexamethasone on sphingomyelin metabolism in nuclear lipid microdomains (NLMs). Sphingomyelin-synthase (**a**) and Sphingomyelinase (**b**) activity was assayed in NLM purified from SUP-T1 cells cultured in the absence (control) or in presence of Dex for different times up to 24 h. The data were expressed as pmol/mg protein/min and represented the mean ± S.D. of three experiments performed in duplicate. (Significance, * *p* < 0.001 *versus* Control sample). Since Dex changed enzyme activities in comparison with control samples at 8 h, ^3^H-palmitic acid incorporation in sphingomyelin and phosphatidylcholine (**c**) was evaluated until to 12 h as reported under “Materials and Methods” section. The data are expressed as cpm/mg protein and represent the mean ± S.D. of 3 experiments performed in duplicate. (Significance, * *p* < 0.001 *versus* control sample).

The incorporation of labelled Dex in cell homogenate, purified Nuclei (N) and NLM was measured at regular intervals of time until to 24 h from Dex incubation and the values were normalized with protein content analyzed in each preparation (Figure 5). Only after 4 h of incubation, ^3^H-Dex was present in the cells with a value about 4 times higher than at 0 h. At the same time, the drug reached the nucleus. The amount of radioactivity/mg protein increased in purified NLM compared to that of the nucleus, indicating that the drug was localized in these specific sites of the inner nuclear membrane [32]. After 8 h the incorporation of ^3^H-Dex into the cells, its transfer into the nucleus and its localization in the NLM was almost complete. In time it is possible to observe a slight and constant increase of radioactivity (Figure 5). In control samples, the activity of SM-synthase showed a peak at 12 h followed by a decrease at 16 h in correspondence with the peak of SMase activity (Figure 6a,b). In the presence of Dex, SM-synthase was higher than control at 8 h, and then slowly decreased, reaching values lower than control at 20 and 24 h. In contrast, Dex induced a decrease in the activity of SMase at 8 h that persisted for the 24 h of incubation (Figure 6a,b). Since SM-synthase is a PC-ceramide PPC transferase which transfers PPC from PC to ceramide for SM synthesis [19], we evaluated the level of ^3^[H] palmitic acid incorporation in PC and SM to analyze the SM behavior within the first 12 h. PC and SM entered into NLM at 4 h and reached a maximum at 6 h (Figure 6c). Dex decreased 62% the level of PC and increased 74% SM between 6 and 8 h (Figure 6c).

## 3. Discussion

It was previously known that the Dex interacts with its receptor in the cytoplasm and then moves to the nucleus where influences gene expression, but there were no information about its intranuclear localization. Our results showed that when Dex enters into the nucleus, it localizes in NLM where stimulates SM-synthase activity with consequent increase of SM content. Previous studies had already shown that SM is preferentially distributed in the perichromatin region which is the major site of DNA replication and transcription and that the microinjection of SMase into nuclei of living cells gives a rapid deterioration of internal nuclear architecture [20]. In fact, the transcriptionally active chromatin fraction is rich in SM content [20] which is characterized by a high amount of saturated fatty acids that favors the formation of Van der Waals interactions with CHO [32]. It is known that the periphery of the nucleus provided a platform for sequestering transcription factors away from chromatin, thus modulating gene expression [33]. We have previously demonstrated that this platform is constituted by SM and CHO to form, together to specific proteins, NLM [16]. When the SM content decreases due to SMase activation, DNA synthesis starts, thus the cells enter in the S phase of the cell cycle [21]. When SM content increases due to SM-synthase activity and links CHO, NLM yields a more rigid structure which regulates transcription processes [23]. Here we show the role of NLM as platform for GC localization and action. It will be interesting in the future to consider if the Dex is present in NLM bound to its receptor with which it is transferred from the cytoplasm, or if it is free or it is bound to a new nuclear receptor. Only after the link to NLM, Dex stimulates the CDKN1A, CDKN1B and GADD45A and reduces STAT3, phosphoSTAT3 and Bcl-2 proteins by inducing delay of cell growth and cell damage. Recent investigations have demonstrated that cytosolic GAPDH is translocated to the nucleus and influences many fundamental cellular functions, including apoptosis, DNA repair, telomere protection, nuclear tRNA export, histone biosynthesis and autophagy [34]. It is possible that the increase of GAPDH expression in our experimental model is due to a metabolic disorder Dex-induced. Some cells change in morphology and others increase in size. It has been demonstrated that Dex treatment of cardiomyocytes causes a significant increase in cell size in the presence of serum and prevents apoptosis triggered by serum depletion [35]. It is possible that in SUP-T1 cells cultured in the presence of serum, Dex has a dual action on the cells; it might stimulate gene expression and activate the protein synthesis for glucidic and lipidic metabolism by increasing cell volume in younger cells and, might activate the apototic machinery in older cells.

At the moment it is not possible to establish whether a damage of the NLM, for example due to an alteration of the lipid component (CHO and SM) following an unbalanced diet, may be responsible for the lack of response of cancer cells to the GC treatment. Further studies will clarify this point.

## 4. Materials and Methods

### 4.1. Materials

Radioactive phosphatidylcholine (PC, l-3-Phosphatidyl *N*-methyl-^3^H choline 1,2 dipalmitoyl, 81.0 Ci/mmol), radioactive SM (choline-metyl ^14^C, 54.5 Ci/mol), radioactive Dex ((1,2,4-^3^H(N))-Dex, 25 mCi/350 pmol) were obtained from Amersham Pharmacia Biotech (Rainham, Essex, UK); Ecoscint A was obtained from National Diagnostic (Atlanta, GA, USA); PC, SM, Non-Hydroxy Fatty Acid Ceramide, Fetal bovine serum (FBS), RPMI 1640 Medium, PSF (penicillin, streptomycin and fungizone) and Dex-water soluble were purchased from Sigma Aldrich Co. (St. Louis, MO, USA). Anti-Bcl-2 antibodies for immunoistochemical analysis were from NOVOCASTRA Laboratories Ltd. Balliol Business Park West (Benton Lane Newcastle, UK). Anti-signal transducer and activator of transcription 3 (STAT3), anti phosphoSTAT3, anti β-actin were obtained from Santa Cruz Biotechnology, Inc. (California, CA, USA); Non-Hodgkin’s T cell human lymphoblastic lymphoma (SUP-T1) were from Biological Materials Bank (ICLC)-Centro Biotecnologie Avanzate (CBA)-Genova, Italy.

### 4.2. Cell Culture and Treatments

SUP-T1 were cultured as previously reported [36]. For each experiments, two lots of cells were prepared: the control sample (C) without Dex and the experimental sample (Dex) with 1.5 μM Dex. To study Dex localization in NLM, 25 μL ^3^H-Dex was diluted with cold Dex to 1.5 μM final concentration and it was added to the culture medium. Cell synchronization was performed as previously reported [37] and the cells were examined at different times from 0 to 48 h.

### 4.3. Preparation of Homogenate, Nuclei and Nuclear Lipid Microdomain Purification

Cells were centrifuged at 1000× *g* for 10 min. The pellets were washed 2× with RPMI 1640 modified medium, centrifuged again at 1000× *g* for 10 min, resuspended in 0.1 M Tris–HCl (Carlo Erba, Milan, Italy) at pH 7.2, and used in part for homogenate analysis and in part for nuclei purification. The homogenate was then used for biochemical determinations and for evaluation of ^3^H-Dex radioactivity. Nuclei were isolated as previously reported [38] and checked for possible cytoplasmic contamination as previously reported [39]. The NLM were prepared in sucrose gradient and checked for possible contamination according to Cascianelli *et al.* [22]. The extraction was carried out with Triton X-100 (Sigma Aldrich Co., St. Louis, MO, USA) dissolved in distilled water (10% *v*/*v*), on ice, added to the purified nuclei to a final detergent concentration of 1% (*v*/*v*).The extract was placed in a cushion of 80% sucrose with a gradient of 15%–40% sucrose on top. After centrifugation overnight, floating fractions corresponding to 3 mL were carefully collected with a pipette, diluted five times with 25 mM HEPES–HCl (Carlo Erba, Milan, Italy), 150 mM NaCl, (Carlo Erba, Milan, Italy) pH 7.1 and centrifuged at 100,000× *g* for 120 min.

### 4.4. ^3^H-Dexamethasone Incorporation

Cells were seeded in 35 flasks and ^3^H-Dex was added to the culture medium as above reported. Cells of 5 flasks were collected at each time points (0, 4, 8, 12, 16, 20 and 24 h), centrifuged at 1000× *g* for 10 min at 4 °C, washed twice and used to prepare cell homogenate, nuclei and NLM. Samples were put into counting vials by adding 10 mL Ecoscint A and 1 mL distilled water. The radioactivity measurements were made with a liquid scintillation analyzer. Data were analyzed in relation to protein content evaluated as reported by Albi *et al.* [21].

### 4.5. Sphingomyelinase and Sphingomyelin-Synthase Activity

For sphingomyelinase (SMase) and sphingomyelin-synthase (SM-synthase) studies the cells were synchronized as above described and cultured with complete medium with or without Dex for 0, 4, 8, 12, 16, 20 and 24 h. At each time point NLM were purified and the enzyme activities were assayed. The SMase and SM-synthase activity was measured in NLM as previously reported [17,18,38].

### 4.6. ^3^H (Sphingomyelin and Phosphatidylcholine) Level

To evaluate the SM or PC behavior, the cells were incubated with 1 µCi/mL of ^3^[H] palmitic acid, diluted with cold palmitic acid to a final concentration of 20 nM in culture medium containing 10% FBS for different times. At each indicated time, the cells from 5 flasks were pooled and NLM were purified. The lipids were extracted and separated on thin layer chromatography (TLC) as reported by Cascianelli *et al.* [22]. The SM and PC spots were scraped and suspended in counting vials with 10 mL Ecoscint A and 1 mL water. The radioactivity was measures in counting vials with 10 mL Ecoscint A and 1 mL water and the radioactivity was measured with a Packard liquid scintillation analyzer (Packard Instrument Company, Meriden, CT, USA).

### 4.7. Electrophoresis and Western Blot Analysis

The level of STAT-3 and phosphoSTAT-3 was analyzed after 24 h of culture. About 30 µg of proteins were submitted to Sodium Dodecyl Sulphate-PolyAcrylamide Gel Electrophoresis (SDS-PAGE electrophoresis, Bio-RAD, Hercules, CA, USA) in 12% polyacrylamide slab gel (Bio-RAD, Hercules, CA, USA) the transfer of protein was carried out into nitrocellulose in 90 min and the immunoblotting was performed by using specific antibodies as reported by Bartoccini *et al.* [24].

### 4.8. DNA Synthesis and Cell Growth

Cells were plated as above reported; 24 h later, cells were washed and synchronized with serum free medium to the G_0_–G_1_ phase of their cell cycle for 24 h. After this time, cells were cultured with complete medium supplemented with 10% FBS and examined at 12, 24, 36 and 48 h. 1 µCi of ^3^H-tymidine was added to the medium 2 h before the analysis. Cells were washed twice with PBS and centrifuged at 800× *g* for 10 min. The pellet was resuspended in 0.1 M Tris, pH 7.6 and used for nuclei purification. The DNA was extracted and used in part for DNA amount determination [38] and in part for radioactivity evaluation as above described for ^3^H-Dex incorporation in NLM. Cell growth was evaluated by counting the number of the cells at regular time intervals, up to 120 h from synchronization.

### 4.9. Morphological and Immunohistochemical Analysis

After 48 h of culture in the presence or absence of Dex, the cells were fixed in 96% ethanol and processed in part for morphological analysis and in part for detection of Bcl-2. For morphological studies the cells were stained with hematoxylin-eosin (Chroma-Gesellschaft, Münster, Germany). Immunohistochemical determination was performed by Bond-max automatic system (Menarini Diagnostics, Firenze, Italy) to detect Bcl-2 as previously reported [36].

### 4.10. Reverse Transcription Quantitative PCR (RTqPCR)

Control and experimental SUP-T1 cells collected after 24 h from Dex treatment were used for total RNA extraction performed by using RNAqueous^®^-4PCR kit (Ambion Inc., Austin, TX, USA). Samples were treated with RNase-free DNase to prevent amplification of genomic DNA possibly present. Samples were dissolved in RNAse-free water and total RNA amount was quantified by measuring the absorbance at 260 nm (A_260_). The purity of RNA was evaluated by using the A_260_/A_280_ ratio. A_260_/A_230_ ratio also was used as indicator of chemical contaminants in nucleic acids. The extracted RNA was immediately frozen and maintained at −80 °C. Before cDNA synthesis, the integrity of RNA was confirmed by 1.2% agarose gel electrophoresis (Invitrogen, Milano, Italy) stained with ethidium bromide (Sigma Aldrich Co., St. Louis, MO, USA). cDNA was synthesized using 1μg total RNA for all samples by High-Capacity cDNA Reverse Transcription kit (Applied Biosystems, Foster City, CA, USA) under the following conditions: 50 °C for 2 min, 95 °C for 10 min, 95 °C for 15 s and 60 °C for 1 min for 40 cycles. RTqPCR was performed using Master Mix TaqMan^®^ Gene Expression and 7.300 RT-PCR instrument (Applied Biosystems), targeting Glyceraldehyde-3-phosphate dehydrogenase (GAPDH; Hs 99999905_m1), CDKN1A (Hs 00355782_m1), CDKN1B (Hs 00153277_m1), B2M (Hs 99999907_m1), GADD45A (Hs 00169255_m1).

### 4.11. Statistical Analysis

Three experiments performed in duplicate where performed for each analysis. Data are expressed as mean ± S.D. and *t*-test was used for statistical analysis.

## 5. Conclusions

When Dex enters into the nucleus of cancer cells, it localizes in NLM to overexpress genes for inhibitor proteins of cell growth.

## References

[1] Moalli P.A., Rosen S.T. (1994). Glucocorticoid receptors and resistance to glucocorticoids in hematologic malignancies. Leuk. Lymphoma.

[2] Schmidt S., Rainer J., Ploner C., Presul E., Riml S., Kofler R. (2004). Glucocorticoid-induced apoptosis and glucocorticoid resistance: molecular mechanisms and clinical relevance. Cell Death Differ..

[3] Laane E., Tamm K.P., Buentke E., Ito K., Kharaziha P., Oscarsson J., Corcoran M., Björklund A.C., Hultenby K., Lundin J. (2009). Cell death induced by dexamethasone in lymphoid leukemia is mediated through initiation of autophagy. Cell Death Differ..

[4] Brunet C.L., Gunby R.H., Benson R.S.P., Hickman J.A., Watson A.J.M., Brady G. (1998). Commitment to cell death measured by loss of clonogenicity is separable from the appearance of apoptotic markers. Cell Death Differ..

[5] Evans R.M. (2005). The nuclear receptor superfamily: A Rosetta stone for physiology. Mol. Endocrinol..

[6] Kininis M., Kraus W.L. (2008). A global view of transcriptional regulation by nuclear receptors: Gene expression, factor localization and DNA sequence analysis. Nucl. Recept. Signal..

[7] Banerjee A., Periyasamy S., Wolf I.M., Hinds T.D.J., Yong W., Shou W., Sanchez E.R. (2008). Control of glucocorticoid and progesterone receptor subcellular localization by the ligand-binding domain is mediated by distinct interactions with tetratricopeptide repeat proteins. Biochemistry.

[8] Burd C.J., Ward J.M., Crusselle-Davis V.J., Kissling G.E., Phadke D., Shah R.R., Archer T.K. (2012). Analysis of chromatin dynamics during glucocorticoid receptor activation. Mol. Cell. Biol..

[9] Vandevyver S., Dejager L., Libert C. (2012). On the trail of the glucocorticoid receptor: Into the nucleus and back. Traffic.

[10] Newbold A., Salmon J.M., Martin B.P., Stanley K., Johnstone R.W. (2013). The role of p21^waf1/cip1^ and p27^Kip1^ in HDACi-mediated tumor cell death and cell cycle arrest in the Eμ-myc model of B-cell lymphoma. Oncogene.

[11] Bustany S., Tchakarska G., Sola B. (2011). Cyclin D1 regulates p27^Kip1^ stability in B cells. Cell Signal..

[12] Moskalev A.A., Smit-McBride Z., Shaposhnikov M.V., Plyusnina E.N., Zhavoronkov A., Budovsky A., Tacutu R., Fraifeld V.E. (2012). Gadd45 proteins: Relevance to aging, longevity and age-related pathologies. Ageing Res. Rev..

[13] Xiong H., Zhang Z.G., Tian X.Q., Sun D.F., Liang Q.C., Zhang Y.J., Lu R., Chen Y.X., Fang J.Y. (2008). Inhibition of JAK1, 2/STAT3 signaling induces apoptosis, cell cycle arrest and reduces tumor cell invasion in colorectal cancer cells. Neoplasia.

[14] Reich N.C. (2013). STATs get their move on. JAKSTAT.

[15] McDonnell T.J., Korsmeyer S.J. (1991). Progression from lymphoid hyperplasia to high-grade malignant lymphoma in mice transgenic for the t(14; 18). Nature.

[16] Albi E., Viola Magni M., Albi E. (2006). Sphingomyelin: A small-big molecule in the nucleus. Sphingolipid and Cell Function.

[17] Albi E., Viola Magni M.P. (1997). Chromatin neutral sphingomyelinase and its role in hepatic regeneration. Biochim. Biophys. Res. Commun..

[18] Albi E., Lazzarini R., Viola Magni M. (2003). Reverse sphingomyelin-synthase in rat liver chromatin. FEBS Lett..

[19] Albi E., Viola Magni M.P. (1999). Sphingomyelin synthase in rat liver nuclear membrane and chromatin. FEBS Lett..

[20] Scassellati C., Albi E., Cmarko D., Tiberi C., Cmarkova J., Bouchet-Marquis C., Verschure P.J., Driel R., Magni M.V., Fakan S. (2010). Intranuclear sphingomyelin is associated with transcriptionally active chromatin and plays a role in nuclear integrity. Biol. Cell.

[21] Albi E., Lazzarini A., Lazzarini R., Floridi A., Damaskopoulou E., Curcio F., Cataldi S. (2013). Nuclear lipid microdomain as place of interaction between sphingomyelin and DNA during liver regeneration. Int. J. Mol. Sci..

[22] Cascianelli G., Villani M., Tosti M., Marini F., Bartoccini E., Viola Magni M., Albi E. (2008). Lipid microdomains in cell nucleus. Mol. Biol. Cell.

[23] Albi E., Villani M. (2009). Nuclear lipid microdomains regulate cell function. Commun. Integr. Biol..

[24] Bartoccini E., Marini F., Damaskopoulou E., Lazzarini R., Cataldi S., Cascianelli G., Gil Garcia M., Albi E. (2011). Nuclear lipid microdomains regulate nuclear vitamin D3 uptake and influence embryonic hippocampal cell differentiation. Mol. Biol. Cell.

[25] El Kadmiri N., Slassi I., el Moutawakil B., Nadifi S., Tadevosyan A., Hachem A., Soukri A. (2014). Glyceraldehyde-3-phosphate dehydrogenase (GAPDH) and Alzheimer’s disease. Pathol. Biol..

[26] Cummings M., Sarveswaran J., Homer-Vanniasinkam S., Burke D., Orsi N.M. (2014). Glyceraldehyde-3-phosphate dehydrogenase is an inappropriate housekeeping gene for normalising gene expression in sepsis. Inflammation.

[27] Li Q.Q., Skinner J., Bennett J.E. (2012). Evaluation of reference genes for real-time quantitative PCR studies in *Candida glabrata* following azole treatment. BMC Mol. Biol..

[28] Schmittgen T.D., Livak K.J. (2008). Analyzing real-time PCR data by the comparative CT method. Nat. Protoc..

[29] Gong J., Luo Y., Zhang Z., Wang W., Li J. (2013). Effect of dexamethasone on expression of interleukin-21 and phospho-STAT3 in a murine model of chronic asthma. J. South Med. Univ..

[30] Clark A.F., Wilson K., McCartney M.D., Miggans S.T., Kunkle M., Howe W. (1994). Glucocorticoid-induced formation of cross-linked actin networks in cultured human trabecular meshwork cells. Investig. Ophthalmol. Vis. Sci..

[31] Sanda T., Tyner J.W., Gutierrez A., Ngo V.N., Glover J., Chang B.H., Yost A., Ma W., Fleischman A.G., Zhou W. (2013). TYK2-STAT1-BCL2 pathway dependence in T-cell acute lymphoblastic leukemia. Cancer Discov..

[32] Albi E. (2011). The role of intranuclear lipids in health and disease. Clin. Lipidol..

[33] Takaoka Y., Goto S., Nakano T., Tseng H.P., Yang S.M., Kawamoto S., Ono K., Chen C.L. (2014). Glyceraldehyde-3-phosphate dehydrogenase (GAPDH) prevents lipopolysaccharide (LPS)-induced, sepsis-related severe acute lung injury in mice. Sci. Rep..

[34] Ren R., Oakley R.H., Cruz-Topete D., Cidlowski J.A. (2012). Dual role for glucocorticoids in cardiomyocyte hypertrophy and apoptosis. Endocrinology.

[35] Heessen S., Fornerod M. (2007). The inner nuclear envelope as a transcription factor resting place. EMBO Rep..

[36] Pugliese L., Bernardini I., Pacifico N., Peverini M., Damaskopoulou E., Cataldi S., Albi E. (2010). Severe hypocholesterolemia is often neglected in hematological malignancies. Eur. J. Cancer.

[37] Marini F., Bartoccini F., Cascianelli G., Voccoli V., Caviglia M.G., Viola Magni M., Albi E. (2010). Effect of 1α,25-Dihydroxyvitamin D3 in embryonic hippocampal cells. Hippocampus.

[38] Albi E., la Porta C.A., Cataldi S., Magni M.V. (2005). Nuclear sphingomyelin-synthase and protein kinase C delta in melanoma cells. Arch. Biochem. Biophys..

[39] Albi E., Cataldi S., Bartoccini E., Magni M.V., Marini F., Mazzoni F., Rainaldi G., Evangelista M., Garcia-Gil M. (2006). Nuclear sphingomyelin pathway in serum deprivation-induced apoptosis of embryonic hippocampal cells. J. Cell Physiol..

